# Assessment of the Effectiveness of Corticosteroid Infiltrations in the Ignace Deen University Hospital Center in Conakry

**DOI:** 10.7759/cureus.49555

**Published:** 2023-11-28

**Authors:** Kaba Condé, Florent Adjakou, Abdoulaye Barry, Moriba Touré, Rosane Livia Fometeu Wouaseha, Fodé Abass Cissé

**Affiliations:** 1 Department of Rheumatology, Ignace Deen University Hospital, Conakry, GIN; 2 Department of Neurology, Ignace Deen University Hospital Center, Conakry, GIN

**Keywords:** musculoskeletal, joint, corticosteroid infiltration, guinea, rheumatic diseases

## Abstract

Introduction

Corticosteroid infiltration is a medical procedure which consists of the injection of a corticosteroid locally, into a painful site. Thus the objective of this study was to evaluate the effectiveness of corticosteroid infiltrations in the rheumatology department of the Ignace Deen University Hospital in Conakry (Guinea).

Patients and method

This was a prospective descriptive and analytical survey that lasted one year from July 2021 to July 2022 carried out in the rheumatology department of the Ignace Deen University Hospital in Conakry. We included all patients who had received corticosteroid infiltration. The infiltration was carried out by a rheumatologist either by anatomical or ultrasound-guided identification.

Results

During the study period, we recorded 1452 observations including 508 (35%) cases of corticosteroid infiltration. The average age was 53.7 years +/- 12.7 years with the majority of our patients were young adults (68%). Females predominated (55%), with a sex ratio (M/F) of 0.80. The infiltrations were mainly indicated for osteoarthritis, particularly knee osteoarthritis in 264 (52%) of cases and lumbarthrosis in 204 (40.2%) of cases, followed by rheumatoid arthritis (RA) in 216 (42.5%) of cases, gout in 183 (36%) of cases and lumbosciatica in 118 (23.2%) of cases. Dexamethasone was the corticosteroid predominantly used in 46.2% of cases. The associated medications were lidocaine in 93% of cases. The intensity of pain remained unchanged in 152 (30%) patients despite the infiltration sessions.

Conclusion

In our study, the majority of patients who benefited from cortisone infiltration were young adults, with females predominating. Osteoarthritis and rheumatoid arthritis were the main indications for infiltration, and dexamethasone was the most commonly used corticosteroid. The study demonstrated the efficacy of cortisone infiltration, with improvement in pain intensity in some patients (70% of cases). For others, however, the pain remained unchanged.

## Introduction

Corticosteroid infiltration is a medical procedure which consists of the injection of a corticosteroid locally, into a painful site [1]. This site can be a joint, a bursa, a synovial sheath, a canal, epidural or peritendinous [1,2]. This analgesic technique, regularly practiced by specialists in rheumatology, known since the 1950s, is part of the therapeutic arsenal and is offered in numerous indications, particularly when it comes to treating a single-joint disorder as in osteoarthritis or pauci-articular outbreaks of inflammatory rheumatism; in abarticular conditions (bursitis, tendinopathy, ligamentitis, chronic nerve compressions) [1,3,4]. The corticosteroids used depend on the joint [1,5,6]. In situ injection of synthetic corticosteroids is effective due to its anti-inflammatory effect [3,7]. The local and systemic effect of such a procedure is difficult to predict, which generates controversy over its indication because the products used have multiple side effects [3,8,9]. Therefore, the objective of this study was to evaluate the effectiveness of corticosteroid infiltrations in the rheumatology department of the Ignace Deen University Hospital in Conakry (Guinea).

## Materials and methods

This was a prospective descriptive study lasting one year, from July 2021 to July 2022, carried out in the rheumatology department of the CHU Ignace Deen in Conakry. This is the only rheumatology department in Guinea for a population of 12 million, and is the reference center for the management of musculoskeletal pathologies. We included all patients with pain who had undergone cortisone infiltration. The infiltration was performed by a rheumatologist either by anatomical location or by ultrasound guidance. Prior to infiltration, aseptic measures were strictly observed (sterile gloves, bib, sterile drape, use of products such as betadine and alcohol). Patients with contraindications (fever, progressive infection, uncontrolled diabetes, malignant hypertension, allergic reactions, rash, psychological disorders) and those lost to follow-up were excluded. For each patient, the following data were collected: sociodemographics (age, sex, marital status, profession and place of origin); clinical: reason for consultation; pain characteristics: type of pain (inflammatory, mechanical, neuropathic), duration of pain (acute: less than or equal to 30 days, subacute: between 30 and 60 days, chronic: greater than or equal to 90 days), site of pain (knee, lumbar, feet, ankles, hip, shoulders, hands/wrists); type of infiltration (anatomical, ultrasound-guided), indications for infiltration: mechanical pathologies (gonarthrosis, lumbar osteoarthritis, coxarthrosis, rhizarthrosis, omarthrosis, lumbosciatica, herniated discs, protruding fingers, cervicobrachial neuralgia); inflammatory pathologies (rheumatoid arthritis, gout, shoulder capsule retraction, lateral epicondylitis, De Quervain's tenosynovitis, Arnold's neuralgia, carpal tunnel syndrome, tarsal tunnel syndrome, rotator cuff tendinopathy); the interval between two infiltrations (one week, three weeks, six weeks, 12 weeks); number of infiltration sessions (two sessions, three sessions); type of corticosteroid used: corticosteroids were used according to their duration of action: short-acting (methylprednisolone acetate), intermediate-acting (triamcinolone acetate, triamcinolone hexacetonide), long-acting (dexamethasone, betamethasone); assessment of pain intensity by visual analog scale (VAS) before and after infiltration, measured on a scale of 1 to 10: 1-3: pain of mild intensity, 3-5: moderate pain intensity, 5-7: intense pain, ˃7: very intense pain; infiltration failure: when pain intensity remains unchanged after two infiltration sessions; drugs associated with corticoids (lidocaine, bupivacaine, hyaluronic acid).

To analyze these data, we performed a descriptive analysis of all the data. Qualitative variables were presented as proportions, and quantitative variables as mean, median, +/- standard deviation. We used Epi Info 7.2 analysis software, developed by the Centers for Disease Control and Prevention (CDC) in Atlanta, Georgia (USA).

All patients signed an informed consent form with the agreement of the CHU Ignace Deen ethics committee, and patient anonymity was preserved.

## Results

**Figure 1:**
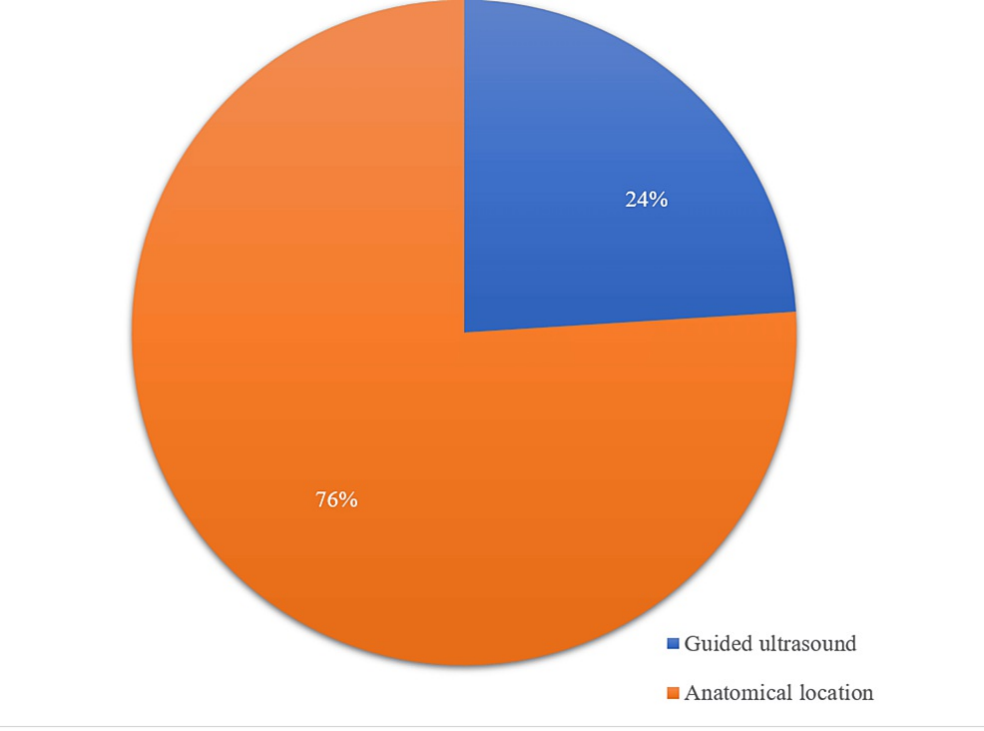
Infiltration technique

**Figure 2:**
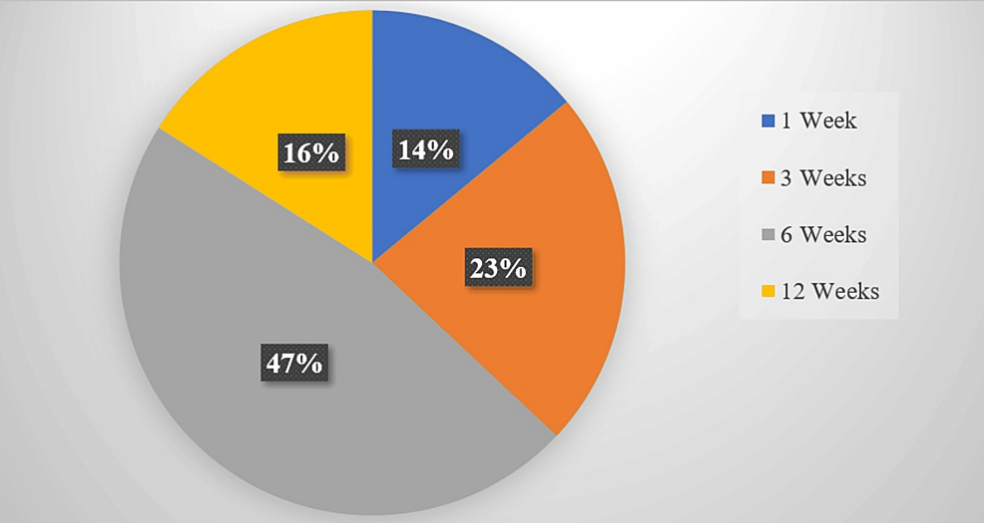
Interval between infiltrations

**Figure 3:**
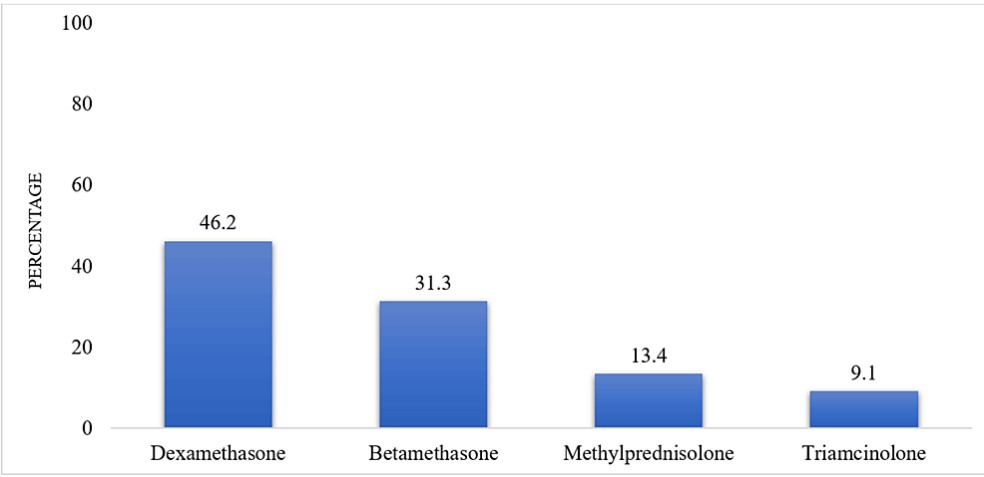
Corticosteroids used

During the study period, we recorded 1452 observations including 508 (35%) cases of corticosteroid infiltration. The average age was 53.7 years +/- 12.7 years (extremes 27 and 86 years) with a female predominance (55%) and a sex ratio (M/F) of 0.80 (Table 1). The reason for consultation was mainly pain in 100% of cases with an average visual analogue scale (VAS) before infiltration of 7 (range of 5 and 9) (Table 2). Pain was inflammatory in 44.1% of cases, with knee pain in 264 (52%) patients (Table 2). The indications for infiltration were dominated by osteoarthritis, particularly gonarthrosis in 264 (52%) of cases and lumbarthrosis in 204 (40.2%) of cases, followed by rheumatoid arthritis (RA) in 216 (42.5%) of cases, gout in 183 (36%) of cases and lumbosciatica in 118 (23.2%) of cases (Table 3). Infiltration by anatomical identification was the most practiced (76%) (Figure 1). Four hundred and sixty-seven patients (92%) benefited from three infiltration sessions (Table 4). The average duration between infiltrations was six weeks (range: 1 to 12 weeks) (Figure 2). Dexamethasone was the corticosteroid most used in 46.2% of cases (Figure 3) in combination with lidocaine (93%) as local anesthesia (Table 4). In osteoarthritis patients, corticosteroids were associated with visco-supplementation with hyaluronic acid in 43%. Despite the infiltration sessions, the intensity of pain remained unchanged in 152 (30%) patients.

**Table 1:** Epidemiological characteristics of patients

Variables	Patients (n = 508)
Average age (Extreme ±) (years)	53.7 (27 and 86)
Sex, female, n (%)	279 (55%)
Marital Status	
Married	345 (67.9%)
Widow/Widower	39 (7.7%)
Single	102 (19.9%)
Divorced	22 (4.5%)
Occupation	
Housekeeper	210 (41.4%)
Driver	161 (31.7%)
Trader	137 (26.9%)
Provenance	
Urban area	361 (71%)
Rural area	147 (29%)

**Table 2:** Pain characteristics MTP: Metatarsophalangeal, VAS: Visual Analog Scale

Variables	Patients n (%)
Type of pain	
Inflammatory	224 (44.1%)
Mechanical	166 (32.7%)
Neuropathic	118 (23.2%)
Duration	
Acute (≤ 30 days)	36 (7.2%)
Subacute (30 to 60 days)	154 (30.3%)
Chronic (≥ 90 days)	318 (62.5%)
VAS before infiltration	
5 – 7	100 (19.7%)
7 – 9	408 (80.3%)
Location	
Knee	264 (52%)
Lumbar	236 (26.7%)
Feet (MTP 1)	186 (36.6%)
Ankle	183 (36%)
Hip	111 (21.8%)
Shoulder	103 (20.3%)
Hand/Wrist	154 (30.3%)

**Table 3:** Indications of infiltration

Indications of infiltration	N (%)
Osteoarthritis	
Gonarthrosis	264 (52%)
Lumbarthrosis	204 (40.2%)
Coxarthrosis	111 (21.8%)
Rhizarthrosis	71 (14%)
Omarthrosis	25 (5%)
Lumbosciatica	118 (23.2%)
Spinal disc herniating	34 (6.7%)
Trigger Finger	15 (2.9%)
Cervicobrachial neuralgia	19 (3.7%)
Rheumatoid arthritis	216 (42.5%)
Gout	183 (36%)
Capsular retraction of the shoulder	56 (11%)
Tennis Elbow	69 (13.5%)
De Quervain’s Tenosynovitis	19 (3.8%)
Occipital Neuralgia	3 (0.5%)
Carpal tunnel syndrome	81 (16%)
Tarsal tunnel syndrome	46 (9%)
Rotator cuff tendinitis	77 (15.1%)

**Table 4:** Data on infiltrations VAS: Visual analog scale

Variables	Patients n (%)
Number of infiltrations	
3 sessions	467 (92%)
2 sessions	41 (8%)
Associated local anesthesia	
Lidocaine	472 (93%)
Bupivacaine	36 (7%)
VAS after infiltration	
1-3	356 (70%)
5-7	152 (30%)

## Discussion

Corticosteroid infiltrations are part of the therapeutic arsenal and are widely used in the daily practice of rheumatologists [4,10]. In our series, 35% of patients seen for one of the indications had benefited from corticosteroid infiltration. Zomalhèto et al. in Benin in 2015 reported a frequency of 17.1% [11]. Nassar et al. in Casablanca in 2014 found a frequency of 62.3% [12]. These data show that corticosteroid infiltrations are frequent practices in hospitals and carried out by the rheumatologist.

The average age of the subjects was 53.7 years with a female predominance and remains close to the data of Zomalhèto et al. who reported 51.15 years. These data demonstrate the predominance of rheumatological conditions, particularly chronic inflammatory rheumatism, in young adult subjects and in women [11]. Pain was the main reason for consultation in all our patients. Indeed, pain is the main symptom of chronic inflammatory rheumatism and abarticular conditions, which generally prompts patients to consult a rheumatologist [13-16].

There are multiple indications for corticosteroid therapy in patients suffering from rheumatic fever. Corticosteroid infiltrations were carried out mainly in patients suffering from osteoarthritis, particularly knee osteoarthritis and lumbar osteoarthritis in our survey. Although osteoarthritis is one of the indications for infiltration, it was less recurrent in other studies which mainly reported rheumatoid arthritis [11,12]. Furthermore, if corticosteroid infiltrations can be carried out regardless of the site of osteoarthritis, in the data available in the literature, infiltrations were carried out mainly in patients suffering from coxarthrosis, rhizarthrosis and omarthrosis [10,17,18].

On the other hand, in our study, infiltrations were performed less in these osteoarthritic sites due firstly to the short-term effectiveness in reducing pain and secondly linked to the fact that infiltrations are not recommended as first intention in certain osteoarthritis sites such as rhizarthrosis [8]. Infiltrations were also carried out in patients suffering from rheumatoid arthritis (RA), gout and lower back sciatica. Zomalhèto et al. reported connective tissue disease, particularly RA and lupus erythematosus, as one of the main indications for the infiltrations found in their study [11]. Nassar et al. found rheumatoid arthritis (50.4%) as the major indication in their series, followed by lupus and vasculitis [12].

Indeed, corticosteroid infiltration into a joint gives rise to the following effects: attenuation of the local inflammatory response by inhibition of the recruitment of inflammatory cells (leukocytes, neutrophils) and mediators of the inflammatory reaction (prostaglandins and interleukins-1); reduction of synovial blood flow and local synthesis of collagen as well as inflammatory cells. All of this helps reduce pain and local inflammation [1]. Also, the divergence of the main indications found in the different studies may be linked to the socio-epidemiological realities of each country.

Anatomical identification was the most used infiltration technique. This is linked on the one hand to the unavailability of ultrasound on a daily basis and on the other hand to the lack of mastery of the ultrasound-guided technique by practitioners. The majority of our patients benefited from three infiltration sessions separated by an interval of six weeks on average between infiltrations. These data are in agreement with those in the literature which recommend not exceeding three to four injections per year for the same joint [1,8].

As for frequency, corticosteroid infiltration depends on the importance of the inflammatory phenomenon, the pathology and the place of injection; for example, osteoarthritis of the knee requires a joint infiltration of corticosteroids to be repeated after six to 12 weeks while tendonitis of the supraspinatus of the shoulder requires a joint infiltration of corticosteroids per week for three weeks [1,19,20].

Furthermore, dexamethasone was the most used corticosteroid associated with lidocaine as local anesthesia. Indeed, the choice of corticosteroid depends on its duration of action (short, intermediate, prolonged), the pathology to be treated and also the availability of the molecules. In our case, the use of dexamethasone was due to its availability and its lower cost as compared to others making it more accessible to patients.

According to literature data, it is recommended to combine an anesthetic substance (lidocaine or bupivacaine) with the corticosteroid compound in order to facilitate the transport and impregnation of the therapeutic substance in the infiltrated tissue. Additionally, this approach quickly alleviates pain (much to the patient's delight) for about an hour or more and thus informs the clinician about the chances of achieving control of the inflammatory response in the longer term [4,6,21].

Visco-supplementation by exogenous injection of hyaluronic acid was carried out only in osteoarthritic patients in our study. Indeed, it aims to restore the basic capacities of the synovial fluid and lubricate the joint so as to absorb shocks and vibrations and reduce the frictional stress of the cartilage [1]. However, not all patients benefited from visco-supplementation in our study and this was linked to the cost and the advanced stage of their osteoarthritis. Despite the infiltration sessions, the pain remained intense in 30% of our patients. This failure of infiltration in certain cases could be due to anatomical location and patients' lack of resources.

The limitations of this study were related both to the patients and to the practicing physicians. These included fear of injections, the cost of infiltration, and patients' inaccessibility to certain molecules. For doctors, lack of training in guided echo was the main obstacle to cortisone infiltration. Training therefore appears essential in the initiation of infiltrations in the future practice of general practitioners and specialists.

## Conclusions

In our study, patients undergoing corticosteroid infiltration were mainly young adults, with females predominating. Osteoarthritis and rheumatoid arthritis were the main indications for infiltration, and dexamethasone was the most commonly used corticosteroid. The study demonstrated the efficacy of corticosteroid infiltration, with an improvement in pain intensity in some patients (356, 70%) of the time. However, for others, pain remained unchanged (30%) of cases.
